# NAMPT reduction‐induced NAD^+^ insufficiency contributes to the compromised oocyte quality from obese mice

**DOI:** 10.1111/acel.13496

**Published:** 2021-10-18

**Authors:** Hengjie Wang, Shuai Zhu, Xinghan Wu, Yuan Liu, Juan Ge, Qiang Wang, Ling Gu

**Affiliations:** ^1^ College of Animal Science & Technology Nanjing Agricultural University Nanjing China; ^2^ State Key Laboratory of Reproductive Medicine Suzhou Municipal Hospital Nanjing Medical University Nanjing China; ^3^ Department of Medical Genetics Maternal and Child Health Hospital of Hunan Province Changsha China

**Keywords:** meiosis, metabolism, nicotinamide phosphoribosyl transferase, obesity, oocyte

## Abstract

Maternal obesity is associated with multiple adverse reproductive outcomes, whereas the underlying molecular mechanisms are still not fully understood. Here, we found the reduced nicotinamide phosphoribosyl transferase (NAMPT) expression and lowered nicotinamide adenine dinucleotide (NAD^+^) content in oocytes from obese mice. Next, by performing morpholino knockdown assay and pharmacological inhibition, we revealed that NAMPT deficiency not only severely disrupts maturational progression and meiotic apparatus, but also induces the metabolic dysfunction in oocytes. Furthermore, overexpression analysis demonstrated that NAMPT insufficiency induced NAD^+^ loss contributes to the compromised developmental potential of oocytes and early embryos from obese mice. Importantly, in vitro supplement and in vivo administration of nicotinic acid (NA) was able to ameliorate the obesity‐associated meiotic defects and oxidative stress in oocytes. Our results indicate a role of NAMPT in modulating oocyte meiosis and metabolism, and uncover the beneficial effects of NA treatment on oocyte quality from obese mice.

## Graphical abstract

This study reports that the potential pathway mediating the effects of NAD+ generation on the quality of HFD oocytes. Loss of NAD+ content and NAMPT protein results in the meiotic defects and oxidative stress in oocytes from obese mice. Nicotinic acid (NA) supplement could partly rescue the defective phenotype of these oocytes.

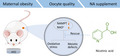

**Abbreviations**
GV: germinal vesicle
GVBD: germinal vesicle breakdown
KD: knockdown
LH: luteinizing hormone
MII: metaphase II
PBEF: pre‐B cell colony enhancing factor
NA: nicotinic acid
NAD+: nicotinamide adenine dinucleotide
NAMPT: nicotinamide phosphoribosyl tranferase
NMNAT: nicotinamide mononucleotide adenylyl transferase
NMN: nicotinamide mononucleotide
NAM: nicotinamide
NR: nicotinamide riboside
Pb1: first polar body
PI: propidium iodide
PMSG: pregnant mare serum gonadotropin
hCG: human chorionic gonadotropin
ROS: reactive oxygen species

## 1 INTRODUCTION

Obesity is the widespread disease among women all around the world (Ng et al., 2014). The amount of women who are distressed about obesity is increasing rapidly. Recent studies stated that women with excessive body fat are often subjected to a series of reproductive problems, such as infertility, miscarriage, and congenital malformations. The effects of obesity on the pregnancy outcome are partially attributed to the changes in oocyte quality. In particular, we have reported that even the metabolic phenotypes of obesity can be reversed, nevertheless, the adverse impact of high‐fed diet on oocyte quality is irreversible (Reynolds et al., 2015). There are emerging data that maternal obesity induces the impaired competence of oocyte, including delayed meiotic progression, mitochondrial dysfunction, and oxidative stress (Hou et al., 2016; Igosheva et al., 2010; Luzzo et al., 2012). Oxidative stress, defined as an imbalance between pro‐oxidant and antioxidant capacity (Hou et al., 2016), is closely related with reactive oxygen species (ROS) synthesis. ROS, a by‐product of oxidative phosphorylation, is dramatically elevated in oocyte from obese females (Han et al., 2017). Excessive amount of ROS can cause serious damage to the cell, and also disturb multiple biological processes (Wang et al., 2018).

Nicotinamide adenine dinucleotide (NAD^+^) is a universal and essential coenzyme found in all species. Interestingly, growing evidence has confirmed that NAD^+^ function not only as a classic cofactor of key enzymes, but also as a multifunctional regulator controlling diverse cell signaling pathways (Yamaguchi & Yoshino, 2017; Yang & Sauve, 2016). Synthesis of NAD^+^ proceeds through multiple pathways. Most tissues synthesize NAD^+^ through the salvage of nicotinamide by nicotinamide phosphoribosyl transferase (NAMPT) (Bowlby et al., 2012). NAMPT, also known as pre‐B cell colony enhancing factor (PBEF) or visfatin, has been implicated in various biological conditions (Imai, 2009). Especially, NAMPT plays a pivotal role in the regulation of cellular metabolism through affecting the activity of NAD‐dependent enzymes (Garten et al., 2015), such as sirtuins and poly(ADP‐ribose) polymerases (Bowlby et al., 2012; Koltai et al., 2010; Yang et al., 2007). In mammals, NAMPT exists in both intracellularly and extracellularly (iNAMPT and eNAMPT, respectively) (Revollo et al., 2007). iNAMPT function has been clarified clearly as an essential NAD^+^ biosynthetic enzyme, while the role of eNAMPT is still obscure. Decline in NAD^+^ level is becoming an established feature of several age‐associated diseases (Covarrubias et al., 2021). To date, however, little information on the connection between NAMPT/NAD^+^ and oocyte quality from obese females is available.

In this study, by employing a high‐fat diet (HFD)‐based mouse model, we discovered a significant reduction of NAMPT protein and NAD^+^ content in oocytes from obese mice. Remarkably, our results show that both in vivo administration and in vitro supplement of nicotinic acid (NA) effectively ameliorate the obesity‐associated meiotic defects and metabolic dysfunction in oocytes.

## 2 RESULTS

### 2.1 Reduced NAMPT expression and NAD^+^ content in oocytes from obese mice

**FIGURE 1:**
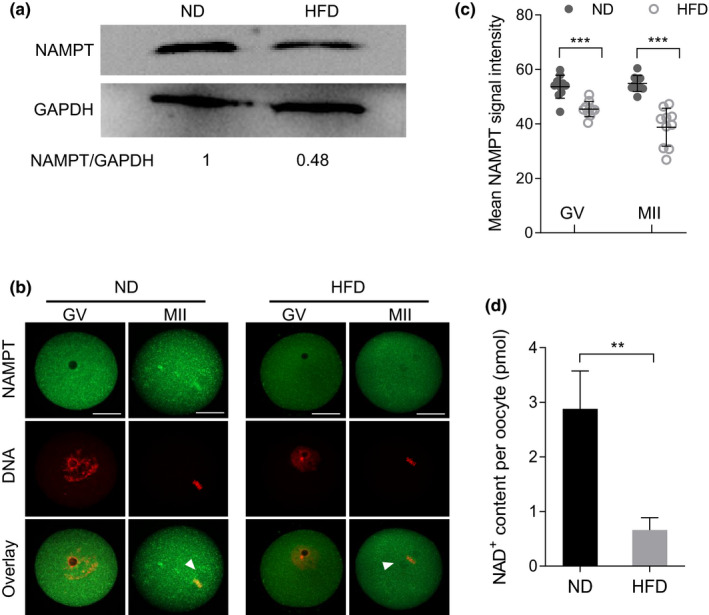
Reduced NAMPT expression and NAD^+^ content in oocytes from HFD mice. (a) Western blotting showing the NAMPT expression in ND and HFD oocytes. GAPDH served as a loading control. Band intensity was calculated using Image J software, the ratio of NAMPT/GAPDH expression was normalized, and values are indicated (200 oocytes per lane). (b) Confocal sections of ND and HFD oocytes stained with NAMPT antibody (green) and counterstained with propidium iodide (red) for DNA. Arrowheads indicate the accumulated NAMPT signal. Scale bars: 30 µm. (c) Quantification of NAMPT immunofluorescence (n = 25 oocytes for ND and HFD). (d) NAD^+^ content in ND and HFD oocytes. Data are expressed as the mean ± SD from three independent experiments. In c, statistical analysis was performed with two‐way ANOVA; and in d, a Student's t test (two‐tailed) was used for statistical analysis. **p < 0.01, ***p < 0.001. HFD, high‐fat diet; NAMPT, nicotinamide phosphoribosyl transferase; ND, normal diet

It has been widely reported that NAMPT accumulation is altered in obesity and obesity‐related disorders (Garten et al., 2015). Hence, we examined whether NAMPT is differentially expressed between oocytes from HFD and normal diet (ND) mice. For brevity, these oocytes are named as “HFD oocytes” and “ND oocytes”, respectively. As shown in Figure 1a, compared to ND oocytes, HFD oocytes displayed a marked reduction in NAMPT protein expression, evidenced by immunoblotting. Consistent with the western blot data, quantitative analysis on the basis of immunostaining also verified that the average fluorescence intensity of NAMPT in HFD oocytes was lower than that in ND cells (Figure 1b‐c). In addition, we noticed that NAMPT resides in entire germinal vesical (GV) oocyte, and then some signals accumulated around the spindle region during meiosis (Figure 1b, arrowheads). NAMPT has been reported to co‐localize strongly with mitochondria which is the energy source of spindle assembly and migration in cells (Wei et al., 2020). Such a specific distribution pattern of NAMPT in oocytes indicates its potential function during meiosis. NAMPT functions as one of the main enzymes responsible for NAD^+^ production. Lastly, we measured the NAD^+^ levels in oocytes, and found that the NAD^+^ content was significantly decreased in HFD oocytes relative to controls (Figure 1d). Collectively, these findings suggest that NAMPT insufficiency induced NAD^+^ reduction may contribute to the compromised quality of HFD oocytes.

### 2.2 NAMPT depletion disrupts meiotic progression and metabolic function in oocytes

**FIGURE 2:**
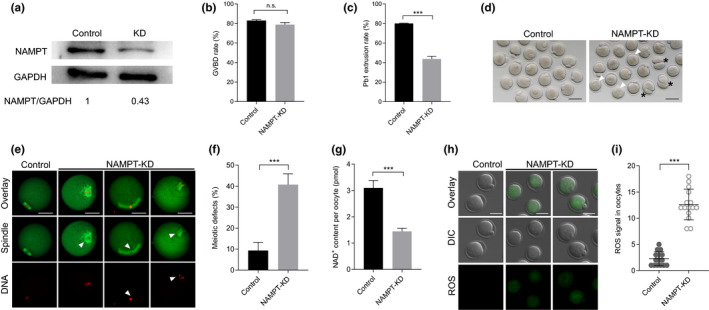
NAMPT depletion disrupts meiotic progression and metabolic function in oocytes. (a) Depletion of NAMPT protein was verified by western blot analysis (200 oocytes per lane). (b,c) Quantitative analysis of GVBD and Pb1 extrusion in controls (n = 115) and NAMPT‐KD (n = 110) oocytes. (d) Bright‐filed images of NAMPT‐KD and control oocytes. White arrowheads indicate the oocytes that fail to extrude polar bodies and black asterisks denote oocytes with aberrant asymmetric division. Scale bars: 80 µm. (e) Representative images of meiotic spindle and chromosomes in control and NAMPT‐KD oocytes. Spindle disorganization and chromosome misalignment are indicated by arrowheads. Scale bars: 30 µm. (f) Quantitative analysis of meiotic defects in control (n = 127) and NAMPT‐KD (n = 103) oocytes. (g) NAD^+^ content in control and NAMPT‐KD oocytes (n = 150 for each group). (h) Representative images of CM‐H2DCFDA fluorescence (green) in oocytes from control and NAMPT‐KD oocytes. Scale bars: 50 µm. (i) Quantification of the levels of ROS in oocytes. Each data point represents an oocyte (n = 15 for each group). Data are expressed as the mean ± SD from three independent experiments. A Student's t test (two‐tailed) was used for statistical analysis. ***p < 0.001. GVBD, germinal vesicle breakdown; NAMPT, nicotinamide phosphoribosyl transferase; NAMPT‐KD, NAMPT protein was knocked down; n.s., not significant; ROS, reactive oxygen species

To test the possibility mentioned above, we first explored the function of NAMPT during oocyte maturation. Fully grown oocytes were microinjected with specifically designed morpholino (MO) in order to sterically block the mRNA translation. About 60% of NAMPT protein was knocked down (NAMPT‐KD) in oocytes as confirmed by immunoblot, while control group injected with a sham MO standard was unaffected (Figure 2a). Both control and NAMPT‐KD oocytes resumed meiosis after 3 h in vitro culture, showing similar germinal vesicle breakdown (GVBD) rate (Figure 2b). However, the ratio of first polar body (Pb1) extrusion was strikingly decreased in NAMPT‐depleted oocytes (Figure 2c). These oocytes exhibited compromised asymmetric division and a high frequency of developmental block (Figure 2d). The failure of meiotic division is often linked with aberrant meiotic apparatus. One of the essential indicators of high‐quality oocytes is normal spindle morphology with aligned chromosomes (Zhang et al., 2019). Therefore, an in‐depth exploration of meiotic apparatus was carried out by immunostaining. Anti‐α‐tubulin antibody was utilized to visualize spindle and chromosomes were counterstained with propidium iodide. In most number of cases, NAMPT‐KD oocytes were assembled with malformed spindle and displaced chromosomes (Figure 2e). The phenotype was about 4 times more prevalent than that in control oocytes which contained typical bipolar spindle and well‐aligned chromosomes (Figure 2f). NAD^+^ was maintained by NAMPT through salvage pathway, so we asked whether NAD^+^ generation was influenced following NAMPT depletion. Consistent with this conception, NAD^+^ levels were reduced by 50% in NAMPT‐KD oocytes compared to controls (Figure 2g). NAD^+^, by allowing the transfer of electrons to produce ATP, serves as a critical cofactor in oxidative phosphorylation (Braidy et al., 2019). Here, by assessing the DCF fluorescence in live cells, we found that ROS levels were drastically increased in NAMPT‐KD oocytes (Figure 2h‐i). Therefore, NAMPT deficiency not only severely compromises maturational progression and meiotic apparatus, but also disrupts the metabolic function in mouse oocytes.

### 2.3 Supplement of nicotinic acid partially rescues the meiotic defects and oxidative stress in oocytes with NAMPT deficiency

**FIGURE 3:**
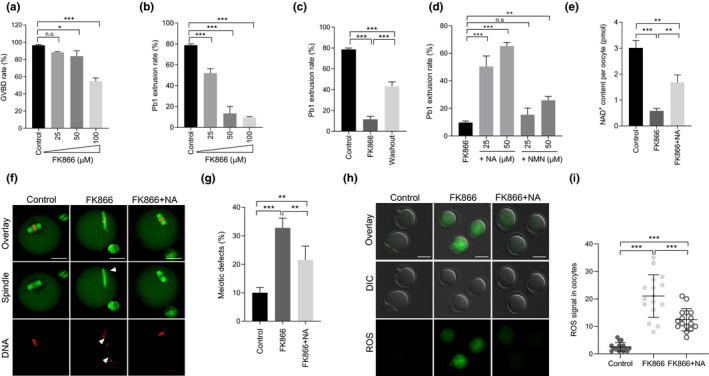
NA supplement partially prevents the meiotic defects and oxidative stress in oocytes with NAMPT deficiency. (a,b) Quantitative analysis of GVBD and Pb1 extrusion in control (n = 138) and FK866‐treated (n = 124 for 25 µM, n = 119 for 50 µM, n = 116 for 100 µM) oocytes. (c) Quantitative analysis of Pb1 extrusion in oocytes after FK866 washout (n = 81 for control, n = 87 for FK866, and n = 73 for washout). (d) Quantitative analysis of Pb1 extrusion in FK866 (n = 93), FK866 + NMN (n = 112), and FK866 + NA (n = 105) oocytes. (e) NAD^+^ content in control, FK866, and FK866 + NA oocytes (n = 150 for each group). (f) Representative images of control, FK866, and FK866 + NA oocytes stained with α‐tubulin antibody to visualize the spindle (green) and with propidium iodide to visualize chromosomes (red). Spindle disorganization and chromosome misalignment are indicated by arrowheads. Scale bars: 30 µm. (g) Quantitative analysis of meiotic defects in control (n = 138), FK866 (n = 124), and FK866 + NA (n = 105) oocytes. (h) Representative images of CM‐H2DCFDA fluorescence (green) in oocytes from control, FK866, and FK866 + NA oocytes. Scale bars: 50 µm. (i) Relative ROS levels in control, FK866, and FK866 + NA oocytes. Each data point represents an oocyte (n = 15 for each group). Data are expressed as the mean ± SD from three independent experiments. Statistical analyses were performed with one‐way ANOVA with Tukey's post hoc test. **p < 0.01, ***p < 0.001. GVBD, germinal vesicle breakdown; n.s., not significant; NA, nicotinic acid; NAMPT, nicotinamide phosphoribosyl transferase

FK866, a low molecular weight compound, could pharmacologically blockade the enzymatic activity of NAMPT (Hasmann & Schemainda, 2003). To determine the effect of NAMPT activity on meiotic maturation, oocytes were cultured in the M16 medium containing various concentrations of FK866 (25, 50 and 100 µM). At the indicated time points (Figure 3a‐b), we checked the ratio of GVBD and Pb1 emission, respectively. Both meiotic resumption and maturation were disrupted by FK866 treatment in a dose‐dependent manner. Accordingly, 100 µM was selected as the optimum concentration for subsequent experiments. It is worth noting that such an inhibitory effect of FK866 was partially reversed through washout experiment (Figure 3c). Emerging evidence has shown that nicotinamide mononucleotide (NMN) or nicotinic acid (NA) supplementation could stimulate NAD^+^ generation and ameliorate the relevant phenotypes (Canto et al., 2015). Here, using FK866‐treated oocytes as a model, we systematically evaluated their protective effects against defective oocyte development. Although both NA and NMN appeared to be able to promote oocyte maturation when NAMPT activity was inhibited (Figure 3d), 50 µM of NA apparently displayed the most significant responsive effects. Similar to NAMPT knockdown, FK866 treatment also resulted in NAD^+^ reduction, meiotic defects, and ROS elevation during maturation (Figure 3e‐i). Of note, all these deficient phenotypes observed in FK866‐treated oocytes were partially rescued by NA supplement in comparison to controls (Figure 3e‐i). Meanwhile, NA treatment was also capable of reducing the abnormalities observed in NAMPT‐KD oocytes, as shown in Figure S1. Together, these results suggest that enzymatic activity of NAMPT is critically required for keeping redox balance and normal meiosis in oocytes. NA, a well‐known precursor of NAD^+^, could function as an antidote for poor oocyte quality due to NAMPT deficiency.

### 2.4 NAMPT overexpression alleviates defective phenotypes of oocytes from obese mice

**FIGURE 4:**
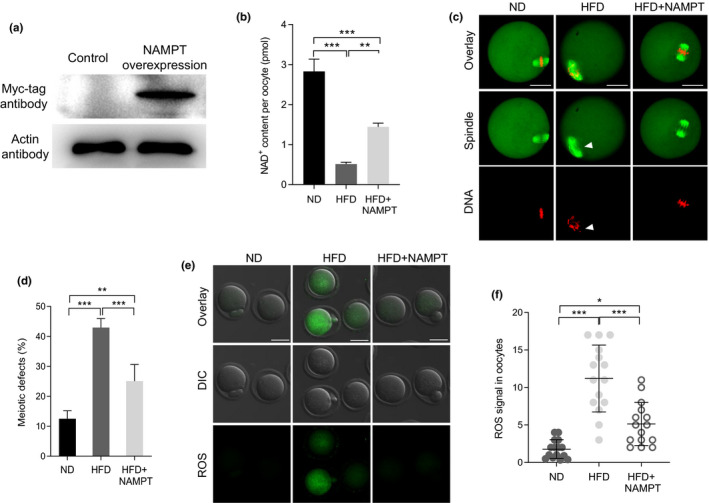
NAMPT overexpression alleviates defective phenotypes of oocytes from obese mice. (a) Immunoblotting showing the overexpression of exogenous NAMPT protein in oocytes (200 oocytes per lane). (b) Quantitative analysis of NAD^+^ content in oocytes of ND, HFD, and HFD + NAMPT (n = 150 for each group). (c) ND, HFD, and HFD + NAMPT oocytes were stained with α‐tubulin antibody to visualize spindle (green) and counterstained with propidium iodide to visualize chromosomes (red). Arrowheads denote the disorganized spindle and misaligned chromosomes. Scale bar: 30 µm. (d) Quantification of ND (n = 102), HFD (n = 119), and HFD + NAMPT (n = 114) oocytes with spindle/chromosome defects. (e) Representative images of CM‐H2DCFDA fluorescence (green) in oocytes from ND, HFD, and HFD + NAMPT oocytes. Scale bars: 50 µm. (f) Quantification of the levels of ROS in oocytes. Each data point represents an oocyte (n = 15 for each group). Data are expressed as the mean ± SD from three independent experiments. Statistical analyses were performed with one‐way ANOVA with Tukey's post hoc test. **p < 0.01, ***p < 0.001. HFD, high‐fat diet; NAMPT, nicotinamide phosphoribosyl transferase; ND, normal diet

Given the reduced levels of NAMPT in HFD oocytes, we hypothesized that introduction of exogenous NAMPT into HFD oocytes may suppress one or more of developmental defects. Toward this goal, we conducted overexpression experiments by injecting *Nampt*‐cRNA into GV oocytes from obese mice. Western blotting verified that exogenous NAMPT protein was expressed in oocytes successfully (Figure 4a). It is noteworthy that ectopic expression of NAMPT in HFD oocytes not only elevated the levels of NAD^+^ (Figure 4b), but also prevented the high occurrence of spindle/chromosome disorganization (Figure 4c‐d) and the excessive generation of ROS (Figure 4e‐f). The results indicate that loss of NAMPT is one of potential factors mediating the effects of maternal obesity on oocyte quality.

### 2.5 In vitro supplementation of NA improves the developmental potential of oocytes and early embryos from obese mice

**FIGURE 5:**
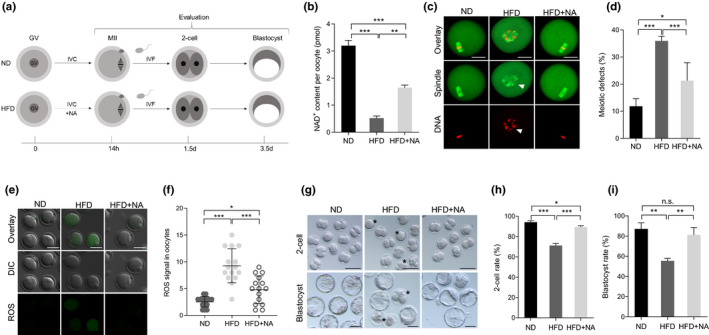
In vitro supplementation of NA improves the developmental potential of oocytes and early embryos from obese mice. (a) The schematic diagram of the experimental procedure. (b) Quantitative analysis of NAD^+^ content in ND, HFD, and HFD + NA oocytes (n = 150 for each group). (c) ND, HFD, and HFD + NA oocytes were stained with α‐tubulin to visualize spindle (green) and counterstained with propidium iodide to visualize chromosomes (red). Representative confocal sections are shown. Arrowheads indicates the disorganized spindle and misaligned chromosomes. Scale bars: 30 μm. (d) Quantification of ND (n = 128), HFD (n = 120), and HFD + NA (n = 103) oocytes with spindle/chromosome defects. (e) Representative images of CM‐H2DCFDA fluorescence (green) in ND, HFD, and HFD + NA oocytes. Scale bar: 50 μm. (f) Quantification of the levels of ROS in oocytes. Each data point represents an oocyte (n = 15 for each group). (g) Representative images of 2‐cell and blastocyst embryos derived from ND, HFD, and HFD + NA oocytes. Asterisks indicate the abnormal HFD embryos. (h‐i) The percentage of embryos that successfully progressed to the 2‐cell and blastocyst stage during in vitro culture (n = 73 for ND, n = 67 for HFD, and n = 67 for HFD + NA). Data are expressed as the mean ± SD from three independent experiments. Statistical analyses were performed with one‐way ANOVA with Tukey's post hoc test. *p < 0.05, **p < 0.01, ***p < 0.001. HFD, high‐fat diet; NA, nicotinic acid; ND, normal diet; ROS, reactive oxygen species

As mentioned above, defective phenotypes in oocytes resulted from NAMPT knockdown or inhibition were rescued through NA treatment. Therefore, we asked whether the developmental competence of HFD oocytes could be improved with NA administration. For this purpose, fully grown GV oocytes were isolated from ND and HFD mice, and then matured in vitro with or without NA (Figure 5a). NA boosted NAD^+^ levels nearly twofold in HFD oocytes relative to their counterparts (Figure 5b). In contrast, the frequency of meiotic defects in HFD oocytes is downregulated by NA from ~40% to ~20% (Figure 5c‐d). Likewise, ROS in HFD oocytes was reduced to nearly normal level following NA administration (Figure 5e‐f). To test whether the developmental capacity of early embryos derived from HFD oocytes could be enhanced via NA supplementation, we next performed in vitro fertilization (IVF) of metaphase II (MII) oocytes, and then early embryos were cultured for further evaluation (Figure 5a). Consistent with previous observation (Han et al., 2018), the formation rate of 2‐cell and blastocyst derived from HFD oocytes were significantly lower than that in ND embryos (Figure 5g‐i), displaying the developmental delay and cytoplasmic fragmentation. However, NA supplement during oocyte maturation markedly increased the proportion of HFD embryos that reached both developmental milestones compared to control embryos.

### 2.6 In vivo administration of NA suppresses meiotic defects and metabolic dysfunctions in oocytes from obese mice

**FIGURE 6:**
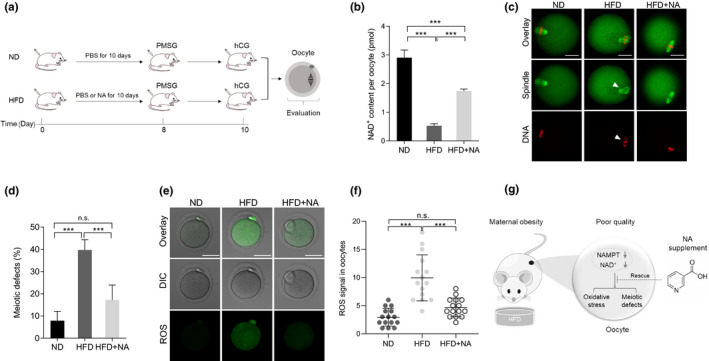
In vivo administration of NA suppresses meiotic defects and metabolic dysfunctions in oocytes from obese mice. (a) A timeline diagram of NA administration and hormone injection. (b) Quantitative analysis of NAD^+^ content in ND, HFD, and HFD + NA oocytes (n = 150 for each group). (c) ND, HFD, and HFD + NA oocytes were stained with α‐tubulin to visualize spindle (green) and counterstained with propidium iodide to visualize chromosomes (red). Representative confocal sections are shown. Arrowheads indicate the disorganized spindle and misaligned chromosomes. Scale bars: 30 μm. (d) Quantification of ND (n = 131), HFD (n = 149), and HFD + NA (n = 122) oocytes with spindle/chromosome defects. (e) Representative images of CM‐H2DCFDA fluorescence (green) in ND, HFD, and HFD + NA oocytes. Scale bar: 50 μm. (f) Quantification of the levels of ROS in oocytes. Each data point represents an oocyte (n = 15 for each group). Data are expressed as the mean ± SD from three independent experiments. Statistical analyses were performed with one‐way ANOVA with Tukey's post hoc test. ***p < 0.001, n.s., not significant. (g) A proposed model showing the potential pathway mediating the effects of NAD^+^ generation on the quality of HFD oocytes. Loss of NAD^+^ content and NAMPT protein results in the meiotic defects and oxidative stress in oocytes from obese mice. NA supplement could partly rescue the defective phenotype of these oocytes. HFD, high‐fat diet; NA, nicotinic acid; NAMPT, nicotinamide phosphoribosyl transferase; ND, normal diet; ROS, reactive oxygen species

Nicotinic acid supplementation in vitro works well. This led us to ask whether artificially replenishing NA in vivo could also exert the protective effects on HFD oocytes. ND or HFD mice were intraperitoneally injected with PBS or NA for 10 days consecutively, and then were received pregnant mare serum gonadotropin (PMSG) on day 8 and human chorionic gonadotropin (hCG) on day 10 for superovulation. Mature oocytes were retrieved to assess the key quality indicators described above (Figure 6a). On the basis of published literatures, we conducted a screening assay (data not shown) to determine the optimal dose for in vivo administration of NA, and 540 mg/kg body weight/day was selected. As shown in Figure 6b‐f, we noted that intraperitoneal administration of NA partly restored the NAD^+^ levels, lowered the frequency of meiotic deficiency, and alleviated the oxidative stress in HFD oocytes. Collectively, these findings suggest that both in vitro supplementation and in vivo administration of NA could improve oocyte quality from HFD mice, and thereupon promote the subsequent embryonic development.

## 3 DISCUSSION

NAD^+^ is an obligate cofactor for the catabolism of metabolic fuels in all cell types (Frederick et al., 2016). It is essential to supply dynamic NAD^+^ turnover permanently for energy‐consuming processes (Canto et al., 2015), which is completed by three biosynthetic pathways: the NAD^+^ de novo pathway, the Preiss‐Handler pathway and the NAD^+^ salvage pathway. Since NAD^+^ contains a nicotinamide (NAM) moiety that cannot be synthesized by most tissues de novo, the vast majority of mammalian cells must instead rely on a salvage pathway to locally regenerate degraded NAD^+^ (Frederick et al., 2016). In this pathway, NAMPT is a determinant of NAD^+^ synthesis and the production of NMN from NAM also relies on NAMPT (Revollo et al., 2007). NAD^+^ can also be synthesized from NA, acid form of vitamin niacin, via Preiss‐Handler pathway in a total of three steps (Fang et al., 2017). A key enzyme of this way is nicotinamide mononucleotide adenylyl transferase (NMNAT), which also involved in the NAD^+^ salvage pathway (Verdin, 2015). Several studies have demonstrated that NA is a more favorable precursor than NAM, in the liver, intestine and kidney (Collins & Chaykin, 1972). Abundant reports about NAMPT physiological functions have recently fueled more enthusiasm to dig potential mechanism in several different fields (Imai, 2009). It has been well documented that NAMPT widely distributes throughout numerous organ systems, in which it plays critical roles in tissue‐specific metabolism. For instance, NAMPT is essential for survival of tumor cells, and is considered a rational target in cancer (Bowlby et al., 2012; Fleischer et al., 2010; Hasmann & Schemainda, 2003; Olesen et al., 2008). A systemic regulatory network, mediated by sirtuins and NAMPT, orchestrates physiological responses to internal and external perturbations (Imai & Yoshino, 2013).

A dynamic balance between production and consumption of NAD^+^ in each subcellular compartment is crucial for pathophysiological process of some diseases (Stein & Imai, 2012). However, the balance can shift during aging when NAD^+^ degradation outrace the ability of cells to synthesize NAD^+^ (Covarrubias et al., 2021). Previous studies have demonstrated that NMNAT2‐NAD^+^‐SIRT1 is an important pathway mediating the effects of maternal age on oocyte developmental competence (Wu et al., 2019). Loss of NAD^+^ biosynthesis in skeletal muscle impairs mitochondrial function and diminishes exercise capacity (Nielsen et al., 2018). NAMPT levels will change so as to cope with metabolism stress (Agerholm et al., 2018), for example, in cases of caloric restriction (Song et al., 2014) or exercise training (Costford et al., 2010; Johnson et al., 2015). Based on these observations, we wondered whether long‐term feeding with high‐fat diet would alter NAMPT expression in germ cells. In the present study, we found that NAMPT accumulation in oocytes recovered from obese mice was markedly declined; and accordingly, the NAD^+^ content was also reduced in these cells. Interestingly, NAMPT depletion or inhibition induced the similar phenotypes as HFD oocytes. Consistent with this observation, Wei et al. demonstrated that NAMPT is involved in the regulation of spindle length and asymmetric division in mammalian oocytes (Wei et al., 2018). Importantly, forced expression of NAMPT was able to partially rescue the phenotypic defects in HFD oocytes. Therefore, these data support the conclusion that loss of NAMPT is an important factor contributing to the compromised oocyte quality of obese mice.

To improve the management of disease caused by lowered NAD^+^ levels, supplementation with either NAD^+^ and its reduced form NADH or its precursors is an ideal therapeutic strategy (Braidy et al., 2019). However, due to a variety of disturbances to absorption and transformation, oral supplementation with NAD^+^ and NADH leads to poor bioavailability (Kimura et al., 2006). Therefore, there is an alternate way, supplementation with NAD^+^ precursors, to get better. Intriguingly, our screening assay clearly showed that, compared to NMN, NA treatment had a more favorable action to correct the NAMPT deficiency‐induced developmental problems in oocytes. NA has been demonstrated to efficiently increase intracellular NAD^+^ levels in brain cells (Grant & Kapoor, 1998). Similarly, NA treatment was reported to attenuate obesity‐induced adipose tissue inflammation (Wanders et al., 2013) and inhibit hepatic lipogenesis in HFD mice (Ye et al., 2020). Moreover, our in vitro and in vivo evidence revealed that boosting NAD^+^ levels by NA supplement to some extent promoted the assembly of meiotic apparatus and cleared the excessive ROS in HFD oocytes, accompanying with the improved developmental potential of preimplantation embryos. To date, however, the detailed pathways controlling NAD^+^ production in mammalian oocytes remain unclear. Clarification of this issue will be important for identifying a better booster of oocyte quality. In addition, whether/how NAD^+^ precursors influence metabolic phenotypes and reproductive outcome of obese mice needs to be systematically evaluated in the future.

The causes of female infertility include anovulation, poor oocyte quality, abnormal fertilization, developmental failure and loss of early embryos (Niringiyumukiza et al., 2018). The underlying mechanisms triggering the reduced female fertility have recently become a focus of intensive investigation. In this study, we provide novel insights into the role of NAMPT during oocyte maturation. In addition, our data highlight the potential therapeutic use of NA to improve oocyte/embryo developmental capability.

## 4 MATERIALS AND METHODS

### 4.1 Mice

Female ICR mice were used in all experiments, and 3 weeks mice were housed in specific pathogen‐free conditions with a 12 h light‐dark cycle. These mice were randomly divided into two diet groups, one group received a HFD (D12492; Research Diets) and the other group received a ND (D1415; Beijing HFK Bioscience) for 16 weeks. After 16 weeks of feeding, body weights (38.3 ± 2.7 g, n = 10 control; 54.3 ± 4.9 g, n = 10 HFD; *p* < 0.05) and fasting serum glucose were significantly higher in mice fed HFD compared with controls. All experimental protocols involving mice were approved by the Animal Care and Use Committee of Nanjing Agricultural University, and all experiments were conducted in compliance with the guidelines of the local animal ethical committee and the Animal Care and Use Committee of Nanjing Agricultural University.

### 4.2 Antibodies

Rabbit polyclonal NAMPT antibody was purchased from Bethyl (Cat#: A300‐372A‐T); Mouse monoclonal FITC‐conjugated anti‐α‐tubulin antibody was purchased from Sigma (Cat#: F2168); mouse monoclonal anti‐Myc tag antibody was purchased from Abcam (Cat#: ab18185); FITC‐conjugated goat anti‐rabbit IgG was purchased from Thermo Fisher Scientific (Cat#: 65‐6111). Except for those specifically stated, all chemicals and culture media in our research were purchased from Sigma.

### 4.3 Collection and culture of oocytes

To collect fully grown GV oocytes, mice were superovulated with an intraperitoneal injection of 5 IU pregnant mare serum gonadotropin (PMSG), and 48 h later, cumulus‐oocyte complexes were obtained by manual rupturing of antral follicles. Cumulus cells were mechanically stripped by repeatedly pipetting and denuded GV oocytes were obtained. For in vitro maturation, oocytes were cultured further in M16 medium at 37°C in an atmosphere of 5% CO_2_ incubator. To collect ovulated MII oocytes, mice were superovulated by injecting 5 IU PMSG followed by 5 IU human chorionic gonadotropin (hCG) 48 h after PMSG priming. 13.5 h post hCG injection, the cumulus‐oocyte complexes were disassociated from the oviducts and digested with 1 mg/ml hyaluronidase incubation. For in vitro supplement, nicotinic acid (N0761, Sigma) was added to maturation medium. For in vivo administration, mice were intraperitoneally injected with nicotinic acid for 10 days consecutively.

### 4.4 Immunofluorescence

As described previously (Liu et al., 2020), oocytes were fixed in 4% paraformaldehyde (PFA) for 30 min, prior to being permeabilized with 0.5% Triton X‐100 for 20 min. After blocking treatment, samples were incubated with primary antibodies at 4°C overnight, and followed with secondary antibodies for 1 h. For spindle examination, oocytes were stained directly with FITC‐conjugated anti‐α‐tubulin antibody (1:200). To detect chromosomes, oocytes were labeled with propidium iodide for 10 min. Finally, oocytes were transferred to a micro‐drop of anti‐fade medium (H1000; Vectashield) on glass slides and observed under a confocal microscope (LSM 710; Carl Zeiss). The fluorescence signal was calculated as the mean intensity (measured from total cytoplasmic intensity and normalized to cell area using ImageJ), following the subtraction of background staining.

### 4.5 Western blotting

Oocytes were washed in ice‐cold PBS before lysed in Laemmli sample buffer with protease inhibitor and boiled for 5 min. Samples were electrophoresed on 10% SDS‐PAGE gel and transferred to PVDF membrane. The membrane was blocked for 1 h with 5% low fat dry milk diluted by PBST at room temperature, and incubated with appropriate primary antibodies overnight at 4°C. After multiple washes, samples were incubated with HRP‐conjugated secondary antibodies. The signal was developed using an ECL Plus Western Blotting Detection System (Thermo Fisher Scientific). GAPDH or Actin was used as a loading control.

### 4.6 ROS evaluation

In order to assess the ROS levels, CM‐H2DCFDA (C6827; Invitrogen) was used. Oocytes were incubated in M16 medium containing with 5 μM CM‐H2DCFDA for 30 min at 37°C in 5% CO_2_ incubator. Following washing three times, oocytes were mounted on a live cell‐imaging dish and covered with mineral oil. Immediately, taking fluorescent images using a Zeiss Laser Scanning Confocal Microscope (LSM 710; Zeiss).

### 4.7 In vitro fertilization and embryo culture

To evaluate the capability of oocyte developmental, we carried out IVF assays according to our previous protocols (Han et al., 2018). Sperm, collected from aged 10–20 weeks male mice, were left to capacitate for 1 h in HTF fertilization medium (MR070; Millipore) supplemented with 10 mg/ml bovine serum albumin, and then co‐incubated with MII oocytes matured in vitro in HTF drops at 37°C for 5 h. Following fertilization, presumptive zygotes were washed in order to remove excess sperm. Finally, zygotes were transferred into KSOM medium (MR106D; Millipore) and cultured up to the blastocyst stage at 37°C in a humidified atmosphere of 5% CO_2_, 5% O_2_, 90% N_2_.

### 4.8 Plasmid construction and mRNA synthesis

Total RNA was extracted from 50 denuded oocytes using the Arcturus PicoPure RNA isolation kit (KIT0204; Applied Biosystems), and cDNA generation was performed using Quantitect Reverse Transcription kit (205311; Qiagen). The following primers were used to amplify the CDS sequence of Nampt:

forward primer, 5′‐GGGGGCCGGCCAGCGGCCGAGATGAATGCT‐3′,

reverse primer, 5′‐GGGCTAGAGGCGCGCCCTAATGAGGTGCCA‐3′.

Purified PCR products were digested with *FseI* and *AscI* (R0558S and R0588S, NEB), and then cloned into the pCS2^+^vector with Myc tags. For the synthesis of Nampt mRNA, the Nampt‐pCS2^+^ plasmids were linearized by *NotI* (R0189S; NEB). The Capped cRNA were made using in vitro transcription with SP6 mMESSAGE mMACHINE (AM1340; Themo Fisher) and purified by RNeasy Micro Kit (74004; Qiagen). Synthesized cRNA was aliquoted and stored at −80°C.

### 4.9 Knockdown and overexpression analysis

Microinjections of morpholino or cRNA were used to knock down or overexpress specific proteins in mouse oocytes, respectively. Ten picoliter cRNA solution (10 ng/µl) was injected into oocyte cytoplasm for overexpression analysis. The same amount of RNase‐free PBS was injected as control. For knockdown experiments, morpholino (MO) of NAMPT (Gene Tools) targeting initiation of translation was diluted with water to give a stock concentration of 1 mM, and then 2.5 pl MO solution was injected into oocytes. NAMPT‐MO: 5′‐CGGCTTCTGCCGCAGCATTCATCTC‐3′; a MO standard control was injected as control. After injections, in order to hinder mRNA translation or facilitate NAMPT overexpression, oocytes were arrested at the GV stage in M16 medium containing 2.5 µM milrinone for 20 h. Following three washes, oocytes were cultured in M16 without milrinone for different time periods to evaluate the cellular events during maturation.

### 4.10 Measurement of NAD^+^ levels

For the NAD^+^ levels, measurements were conducted by a commercially available kit (MAK037; Sigma) as previous described (Wu et al., 2019). 150 oocytes were harvested for total NAD^+^ extraction and quantified as manufacture's instruction. The NAD^+^ concentration was calculated by subtracting the NADH values from NAD_total_ (NAD^+^ and NADH). The NAD^+^ content of samples was quantified with a plate reader in a colorimetric assay at 450 nm using iMark™ Microplate Absorbance Reader (BIO‐RAD).

### 4.11 Statistical analysis

Data are expressed as means ± SD, unless otherwise stated. All analyses were performed using GraphPad Prism (Version 7.0) for Windows. Statistical comparisons were made with two‐tailed Student's t test, two‐way ANOVA, and one‐way ANOVA test when appropriate. Changes were considered statistically significant when *p* < 0.05.

## CONFLICT OF INTEREST

The authors have nothing to disclose.

## AUTHOR CONTRIBUTIONS

HW, LG, and QW designed research. HW, SZ, XW, YL, and JG performed research. HW, SZ, LG, and QW analyzed data. HW, LG, and QW wrote paper.

## Supporting information

Figure S1

## Data Availability

The data that support the findings of this study are available from the corresponding author upon reasonable request.
