# Supplementary material for: NAMPT reduction‐induced NAD+ insufficiency contributes to the compromised oocyte quality from obese mice
Source: Aging Cell. 2021 Oct 18;20(11):e13496. doi: 10.1111/acel.13496 (PMC8590097; doi:10.1111/acel.13496)
Supplement: Supplementary file 1 — Figure S1 [file ACEL-20-e13496-s001.pdf]

Figure S1

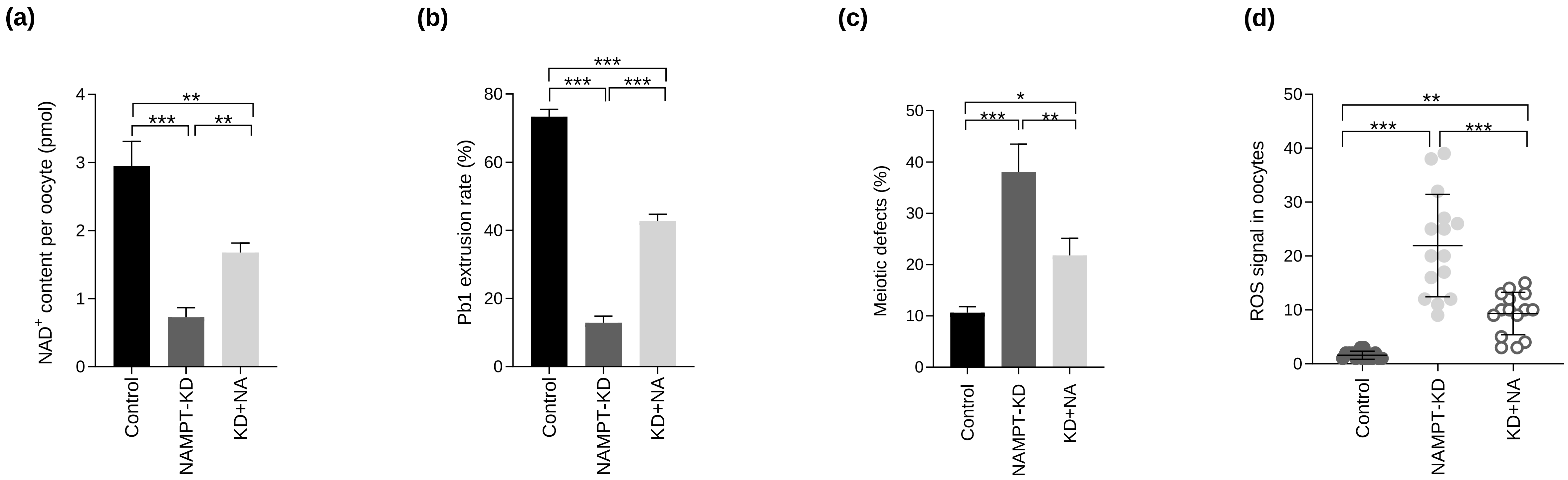

Figure S1. NA supplement partially suppresses the meiotic defects and oxidative stress caused by NAMPT knockdown. (a) Quantitative analysis of NAD^+ content in oocytes of control, NAMPT-KD, and NAMPT-KD+NA (n=150 for each group). (b) Quantitative analysis of Pb1 extrusion in control (n=94), NAMPT-KD (n=97), and NAMPT-KD+NA (n=89) oocytes. (c) Quantitative analysis of meiotic defects in control (n=90), NAMPT-KD (n=92), and NAMPT-KD+NA (n=88) oocytes. (d) Quantification of the levels of ROS in oocytes. Each data point represents an oocyte (n=15 for each group). Data are expressed as the mean ± SD from three independent experiments. Statistical analyses were performed with one-way ANOVA with Tukey's post hoc test. *P<0.05, **P<0.01, ***P<0.001.
